# Personality functioning improvements in adolescents from an early intervention clinic for personality disorders

**DOI:** 10.1007/s00787-025-02913-4

**Published:** 2025-11-10

**Authors:** Luana Palermo, Marialuisa Cavelti, Silvano Sele, Carla Sharp, Corinna Reichl, Michael Kaess

**Affiliations:** 1 University Hospital of Child and Adolescent Psychiatry and Psychotherapy, University of Bern, Bern, Switzerland; 2 Graduate School for Health Sciences, University of Bern, Bern, Switzerland; 3 Department of Psychology, University of Houston, Houston, TX USA; 4 Department of Child and Adolescent Psychiatry, Centre for Psychosocial Medicine, University Hospital Heidelberg, Heidelberg, Germany

**Keywords:** Personality Disorder, Personality Functioning, Adolescence, Early Detection, Mixed-Regression Models

## Abstract

Early intervention of personality disorders (PD) in adolescents is crucial due to their long-term developmental impact. Dimensional models, like the DSM-5 Alternative Model for Personality Disorders (AMPD), may facilitate early detection and intervention, although their applicability in adolescent clinical populations is under-researched. This longitudinal study examines changes in PD diagnosis and impairments in personality functioning according to the AMPD and explores potential predictors in a clinical adolescent cohort. 227 adolescents (*M* age = 15.25, 85.02% female) from an outpatient clinic for early PD intervention were assessed at baseline, one year, and two years. Personality functioning was measured using the Semi-Structured Interview for Personality Functioning, psychiatric diagnoses with the Mini International Neuropsychological Interview, and psychosocial functioning with the Social and Occupational Functioning Assessment Scale. Mixed regression models were applied for analysis. The probability of a PD diagnosis significantly decreased over two years (OR = 0.54, p = .011). Significant improvements were observed in identity (β = -0.191, *p* < .001), self-direction (β = -0.243, *p* < .001), empathy (β = -0.147, *p* < .001) and intimacy (β = -0.136, *p* < .001). Age, sex, psychiatric comorbidity, psychosocial functioning at baseline, treatment setting (inpatient versus outpatient) and dose did not influence PD diagnosis remission or changes in personality functioning, but higher baseline psychosocial functioning was linked to less improvement in self-direction (β = 0.008, *p* = .033). Personality functioning improved over time, supporting the notion that PD pathology in adolescents is amenable to change. Further research is needed to examine individual trajectories of personality functioning and refine targeted intervention strategies.

**Supplementary Information:**

The online version contains supplementary material available at 10.1007/s00787-025-02913-4.

## Introduction

Personality Disorders (PD) are serious mental disorders affecting 1–3% of adolescents in the general population [1], and up to 50% of young inpatients [2, 3]. Adolescents with PD have high rates of co-occurring psychiatric disorders [2, 4, 5], report low quality of life, and generate high health care costs [6]. They engage in more health-risk behaviours such as smoking, alcohol and substance misuse and risky sexual activity [7] and exhibit more delinquent behaviour than adolescents without PD symptoms [8]. Furthermore, PD in youth have been shown to predict several negative outcomes in adulthood including diminished life satisfaction, impaired mental health, poor academic and occupational attainment, problems in social support and relationships, and poor residential, mobility and financial and health resources [8, 9]. Therefore, it is not surprising that early detection and intervention of PD in adolescents has recently become a global health priority [10].

While there appears to be scientific consensus regarding the importance of early diagnosis and treatment of PD in young individuals [11–13], the optimal conceptualization remains a topic of debate. Indeed, the traditional categorical approach utilized for diagnosing PD has faced widespread criticism due to several shortcomings like high rates of comorbidity among PD, the reliance on arbitrary thresholds, the substantial heterogeneity within PD categories, the high prevalence of “personality disorders not otherwise specified”, and the limited clinical utility in predicting treatment efficacy [14–16]. To overcome these limitations the field has witnessed a shift to dimensional conceptualizations of PD, such as the Alternative Model for Personality Disorders (AMPD) of the fifth revision of the Diagnostic and Statistical Manual of Mental Disorders (DSM-5) [17]. The dimensional approach moves away from the either/or-approach, and rather considers personality pathology on a continuum, ranging from none to severe personality pathology. This approach may provide more nuance, enabling the identification of young individuals that are at risk or at earlier stages of developing PD [18]. The AMPD conceptualizes PD through two criteria: The first encompasses the Level of personality functioning (LoPF) – Criterion A – and defines impairments in self- (identity and self-direction) and interpersonal (empathy and intimacy) functioning as the shared core of all PD. The second component includes maladaptive personality traits – Criterion B – organized into five domains: detachment, antagonism, negative affectivity, disinhibition and psychoticism. Although (maladaptive) personality traits are already observable in early childhood, a diagnosis of PD is typically not assigned until adolescence [19]. This is because certain cognitive and neural functions, which become crucial during adolescence, undergo qualitative changes that are necessary for managing new adult roles and responsibilities [20]. As the developmental tasks during adolescence encounter heightened demands in self- and interpersonal functioning (e.g., identity formation, establishing intimate relationships), the symptoms of PD can become more pronounced in this age. For this reason, Criterion A—rather than Criterion B—is argued to account for the onset of personality pathology in adolescence [21]. Accordingly, adolescence is considered a window of opportunity for early detection and intervention of PD [19], and Criterion A may represent both a more developmentally appropriate way to assess personality pathology in young people and a suitable target for intervention.

Previous research on Criterion A in adolescence underscores its clinical utility [18, 22, 23]. Indeed, studies have shown that Criterion A is linked to general psychiatric severity, diverse domains of psychopathology, and adaptive functioning [24, 25], can distinguish between adolescents with and without PD [26] and provides important insights beyond severity that can inform treatment planning [27]. Despite the growing body of evidence supporting Criterion A, little is known about the long-term course of personality functioning (PF) impairments and how these impairments respond to treatment. To date, it has been shown that PD diagnoses are not intractable and interventions such as Dialectic Behavioral Therapy (DBT) or Mentalization-based treatment (MBT) can significantly reduce PD symptoms [12, 28]. With regards to PF, one longitudinal study on adult patients receiving PD specific care reported that PF (as assessed by the Levels of PF Scale—Brief Form; LPFS-BF) improved over an average period of 20 months [29]. In addition, in a clinical youth sample aged 12–26 years, Iannattone and colleagues (2024) observed that PF (as assessed by the LPFS-BF) fluctuated across three measurements within one year. These fluctuations were attributed to social and physical challenges inherent to this developmental period, as well as potential treatment effects. However, no study has yet investigated whether the different aspects of PF (i.e., identity, self-direction, empathy, and intimacy) change in a similar or different manner over time. This is an important question to address, as an advantage of the AMPD is that it allows the identification of specific areas of personality dysfunction, fostering more targeted and individualized intervention strategies. Additionally, the few available longitudinal studies on personality dysfunction have relied exclusively on self-reports. However, clinician evaluations are needed for more rigorous assessment, which is particularly important given the complexity of the construct of PF and the difficulties this poses for accurate self-assessment. Finally, between-person differences that can influence the course of PF impairments in patients are not well understood, although they may enable interventions to be tailored to specific subgroups. For example, one study on youth patients has found greater impairment in PF among females compared to males over time [30]. Moreover, previous studies utilizing the traditional categorical diagnostic approach suggest that age [31], psychiatric severity [32, 33], psychosocial functioning [34], treatment setting (outpatient versus inpatient) [35] and dose [36, 37] can influence the remission of PD diagnosis and symptoms. However, no study has yet examined these potential moderating effects on changes in personality dysfunction among adolescents.

To address these gaps, this study aims to examine 1) remission of PD diagnosis according to the AMPD (categorical), and 2) changes in the degree of impairments in identity, self-direction, empathy and intimacy (dimensional)—including potential differences in the rates of improvement across these elements—over two years in an adolescent patient cohort recruited from an early intervention clinic for PD. Based on what was observed in traditional categorical PD and in the few studies available on PF [12, 28–30], we hypothesize that PD will remit and personality dysfunction will improve over time. Additionally, this study aims to 3) explore factors that potentially predict the likelihood of PD diagnosis remission (3a) and changes in impairments in identity, self-direction, empathy, and intimacy (3b), including age, sex, psychiatric comorbidity, functional impairments, treatment setting (i.e., outpatient versus inpatient), and dose (i.e., numbers of outpatient therapy sessions or inpatient days, respectively). Based on previous literature, we assume that females will show less improvement in PF [30], and that higher age [31], lower psychiatric comorbidity [32, 33], and higher psychosocial functioning at baseline [34], as well as outpatient treatment (compared to inpatient treatment) [35], and a higher treatment dose [36, 37], will be associated with greater improvement in personality dysfunction.

## Methods

### Participants and procedure

Participants were consecutively recruited between 2019 and 2023 from the AtR!Sk clinic *(Ambulatorium für Risikoverhalten und Selbstschädigung)* at the University Hospital of Child and Adolescent Psychiatry and Psychotherapy in Bern, Switzerland. AtR!Sk is a specialized outpatient clinic for early intervention for Borderline Personality Disorder (BPD), offering consultation, diagnostics and therapy for adolescents aged 12 to 17 years who show risky or self-harming behaviour [38]. After a diagnostic assessment at clinic entry, patients are offered a manualized, 10-session cognitive-behavioural intervention for non-suicidal self-injury (NSSI), the Cutting Down Program (CDP) [39, 40]. Patients with persistent BPD symptoms (i.e., three or more BPD criteria in the Structured Clinical Interview for DSM-IV-Axis II Personality Disorders [SCID-II] [41, 42] and an overall severity score of 6 or higher in the Zanarini Rating Scale for BPD [ZAN-BPD] [43] after CDP are then offered Dialectical Behavioural Therapy for Adolescents (DBT-A), including 25 individual and 20 group therapy sessions. During both CDP and DBT-A, patients receive additional family therapy sessions, psychiatric management, and specialist crisis involvement when necessary.

At the diagnostic assessment at clinic entry, patients were informed about the study. Inclusion criteria for the study were: 1) 12-17 years of age, 2) occurrence of at least one risky or self-harming behaviour like NSSI, past suicide attempt, excessive alcohol consumption and/or substance abuse, excessive media or internet use, sexual risk behaviour, or delinquent behaviour, and 3) completion of baseline diagnostics. All participants or their legal guardians (if under the age of 14) provided written informed consent before inclusion in the study. The baseline assessment (T0) was part of the routine clinical procedure. Further assessments were conducted one year (T1) and two years (T2) after baseline and were reimbursed with 85 Swiss Francs each. The assessments were carried out by trained clinicians (psychologists or physicians) or PhD students. All raters underwent a standardized training program conducted by an experienced diagnostician. Training included (a) observation of assessments rated by experienced psychologists or PhD students already trained in the instrument, (b) joint ratings with these experienced raters, and (c) independent ratings under direct supervision. Throughout the data collection period, raters participated in regular supervision meetings to discuss ratings and resolve potential ambiguities. The AtR!Sk cohort study was conducted in accordance with the Declaration of Helsinki [44] and approved by the Swiss Ethic Committees on research involving humans (ID 2018–00942). This study was not preregistered. 

### Measures

#### Demographic data

Sociodemographic information was collected using a standardized set of interview questions, assessing age, biological sex, school type, family and living situation.

#### Personality dysfunction

Personality dysfunction was assessed using the Semi-Structured Interview for Personality Functioning DSM-5 (STiP-5.1) [45]. The STiP-5.1 consists of two PF domains: self and interpersonal. These domains are further subdivided into four elements: identity and self-direction, empathy and intimacy. For each element, clinicians rate 3 items (facets) using a scale from 0 to 4 (with 0 = no impairment, 1 = some impairment, 2 = moderate impairment, 3 = severe impairment and 4 = extreme impairment). The interview comprises 28 open questions and takes between 45 and 60 minutes. The STiP-5.1 has high internal consistency (Cronbach’s α = 0.97) and high interrater reliability (interclass correlations ICC ranging from 0.81 to 0.92) [45]. The German version also exhibits good inter-rater reliability (ICC = 0.93) [46] and the STiP-5.1 shows appropriate psychometric properties also in adolescents [18]. In the present cohort study, interrater reliability was evaluated for 29 patients by two raters and the ICC for the STiP-5.1 total score was high (ICC = 0.841). The diagnostic threshold for a PD diagnosis was defined as having a score of two or higher on two or more elements according to the AMPD guidance for trait-specified and specific PD.

#### Psychiatric diagnoses

Psychiatric diagnoses were measured using the German version of the Mini International Neuropsychiatric Interview for Children and Adolescents (MINI-KID 6.0). The Mini-KID is based on the DSM-IV and the International Classification of Diseases (ICD-10) and is a short, structured interview with good psychometric properties [47], including good area under the curve (AUC) for interrater reliability of ≥ 0.89 [48]. In the current study, the diagnoses were grouped into categories according to the ICD-10 (i.e., F10-F90).

#### Psychosocial functioning

Functional level was evaluated through the Social and Occupational Functioning Assessment Scale (SOFAS) [49]. This clinician-rated measure ranges from 0 (lowest functioning level) to 100 (highest functioning level), whereby each 10 points are combined to form a level. The SOFAS evaluates social and occupational functioning regardless of the overall severity of the individual’s psychopathological symptoms. It has good validity and reliability [50], including high interrater reliability (ICC = 0.86) [51].

#### Treatment setting and dose

Both outpatient and inpatient treatment were assessed retrospectively at each follow-up. Participants reported the number of days they had spent in an inpatient ward, and/or the number of outpatient therapy sessions they had attended in the past year, respectively. For the operationalization, we summed the number of outpatient sessions and separately, the number of inpatient days reported at follow-up 1 and follow-up 2 to obtain a cumulative dose across the study period.

### Statistical analyses

To compare participants who only took part at baseline with those who participated at baseline and at least one follow-up, we examined differences in PF, age, sex, psychiatric diagnoses, and psychosocial functioning. Mann–Whitney U Tests were conducted for continuous variables, while Chi-square (χ2) tests were used for categorical variables.

To examine if the PD diagnosis according to the AMPD changes over time, mixed-effects logistic regression was conducted. PD diagnosis (yes, no) was used as a binary outcome variable. Time (baseline (0), 1-year follow-up (1), and 2-year follow-up (2)) was modelled as a continuous variable, and entered as a fixed effect, while the subjects’ ID was entered as a random effect into the model. To examine whether age (years), sex (male, female), psychiatric comorbidity (each F10 to F90 category yes/no), and psychosocial functioning (highest SOFAS score in the past month) at baseline, and inpatient treatment dose (i.e., total days of inpatient treatment) and outpatient treatment dose (i.e., the number of outpatient therapy sessions) over time affects the change in PD diagnosis over time, we conducted mixed-effects logistic regressions. Each model included the predictor variable and the interaction term “predictor x time” as fixed effects. We tested four separate models, focusing each on a different set of predictor(s): (1) age and sex, (2) psychiatric comorbidity, (3) psychosocial functioning, and (4) treatment setting and doses. Age and sex were considered as covariates in model 2 and 3. In model 4, the covariates expanded to include age, sex, psychiatric diagnoses, and psychosocial functioning in order to account for baseline differences in clinical severity. For models 1, 2, and 4, which included more than one predictor variable each, a Likelihood-Ratio Test (LRT) was performed in order to determine whether the inclusion of the interaction terms improves the model fit compared to the null model including the main effect only. A significant LRT indicates that the predictor influences PD remission over time and in this case, the alternative model including the interaction term is presented. If the LRT is non-significant, there is no evidence that the predictor affects PD remission and the null model with only main effects is presented.

To test whether impairments in identity, self-direction, empathy and intimacy decreased over time mixed-effects linear regressions were conducted, with the mean of each STiP-5.1 element (calculated from the respective facets) as outcome variable separately. Again, time was modelled as continuous variable and entered as a fixed effect, while the subjects’ ID was entered as a random effect into the model. Additionally, a random intercept and a random slope were included for the variable “time” for each subject.

To compare the average changes between the different elements, we fitted a multivariate latent growth curve model (LGCM) with random intercepts and slopes for each element. The model accounted for correlations between elements by allowing random effects (i.e., intercept and slopes) to be correlated. Additionally, residuals at the same time points were allowed to correlate. Measurement error variances and covariances were constrained to be equal within sets of observed variables. After conducting multivariate LGCM, the Wald test was employed, to test if the average slopes differed between the elements.

Finally, the testing of possible predictive effects of age, sex, psychiatric comorbidity, psychosocial functioning, and treatment setting and dose on the degree of change in the elements over time followed the same procedure as described above.

All analysis were conducted in Stata (version 18.0) and significance level was set at α = 0.05.

## Results

### Sample characteristic

Two-hundred and twenty-eight adolescents completed the baseline assessment and provided written informed consent to participate in the study. Out of these, one individual was excluded because the STiP-5.1 at baseline was not completed. The final sample was *N* = 227, of which 118 (52% of the baseline sample) took part at follow-up 1 and 87 (74% of the follow-up 1 sample) at follow-up 2. The mean age at baseline was 15.25 years (SD = 1.48) and 193 (85.02%) patients were female. 56 adolescents (24.67%) met diagnostic threshold for PD according to DSM-5 AMPD guidance of trait-specified and specific PD. Further participants’ characteristics are shown in Table 1.

**Table 1:** Sample characteristic

	Baseline (*n* = 227)	Follow-up 1 (*n* = 118)	Follow-up 2 (*n* = 87)
**Family status n(%)**			
Living together	106 (46.70)		
Parents separated/divorced	115 (50.66)		
Parents separated by death	2 (0.88)		
Parents never lived together	3 (1.32)		
Unknown/other	1 (0.44)		
School type n(%)			
No school-leaving qualification	7 (3.08)		
Primary school (ISCED levels 0–1; at least 6 school years)	23 (10.13)		
Secondary school (ISCED level 2; 9–10 school years)	166 (73.13)		
High school (ISCED level 3; 12–13 school years)	28 (12.33)		
Other/no information	3 (1.32)		
**Diagnoses**^**1**^ **n(%)**			
F1 Mental and behavioral disorders due to psychoactive substance use	77 (33.92)		
F2 Schizophrenia, schizotypal and delusional disorders	32 (14.22)		
F3 Affective disorder	138 (61.06)		
F4 Neurotic, stress-related and somatoform disorders	141 (62.39)		
F5 Behavioural syndromes associated with physiological disturbances and physical factors	21 (9.29)		
F6 Personality and behavioural disorders^2^	40 (17.62)		
F9 Behavioural and emotional disorders with onset in childhood and adolescence	100 (44.25)		
**STiP-5.1 M(SD)**			
Total score	1.13 (0.66)	0.90 (0.73)	0.73 (0.72)
Identity	1.60 (0.85)	1.41 (1.16)	1.15 (1.07)
Self-direction	1.27 (0.98)	0.87 (0.85)	0.79 (0.92)
Empathy	0.83 (0.72)	0.65 (0.71)	0.51 (0.61)
Intimacy	0.82 (0.76)	0.67 (0.77)	0.49 (0.81)
PD n(%)	56 (24.67)	20 (16.95)	12 (13.79)
**Treatment** **n(%)**			
Number of participants with inpatient treatment in the past year^3^		29 (26.13)	20 (25.64)
Of those, inpatient days in the past year M(SD)		13.12 (38.93)	21.67 (52.64)
Number of participants with outpatient treatment in the past year^4^		94 (80.34)	57 (65.52)
Of those, outpatient sessions attended in the past year M(SD)^5^		19.5 (13.32)	31 (27.92)

M= mean, SD= standard deviation, n= sample, ISCED= International Standard Classification of Education STiP-5.1 = Semi-Structured Interview for Personality Functioning, PD = Personality Disorder

^1^F0 (organic mental disorders), F7 (mental retardation) and F8 (disorders of psychological development) were not assessed. Comorbidity was possible. Two patients had missing values in each F category.

^2^F6 is represented by Borderline Personality Disorder assessed with the Structured Interview for DSM-5 Borderline Personality Disorder

^3^There were some missing values and one patient was excluded because reported inpatient clinic was not a psychiatric facility: For Follow-up 1 n = 111, for Follow-up 2 n = 78

^4^There was one missing value in Follow-up 1, n = 117

^5^There was one missing value in Follow-up 2, n = 56

Participants who only completed the baseline assessment did not differ significantly from those who participated in baseline and at least one follow-up assessment in terms of impairments in identity (z = 0.629, *p* = 0.530), self-direction (z = 0.962, *p* = 0.336), empathy (z = −0.511, *p* = 0.609), intimacy (z = 0.377, *p* = 0.706), PD diagnosis according to the AMDP (χ2 = 0.063, *p* = 0.802), age (z = 1.239, *p* = 0.215), sex (χ2 = 0.716, *p* = 0.699), ICD-10 diagnoses (F1: χ2 = 0.023, *p* = 0.880, F2: χ2 = 1.190, *p* = 0.275, F3: χ2 = 0.085, *p* = 0.771, F4: χ2 = 0.639, *p* = 0.424, F5: χ2 = 0.029, *p* = 0.865, F9: χ2 = 0.597, *p* = 0.440), and psychosocial functioning (z = 0.808, *p* = 0.420).

### Longitudinal change in personality disorder (PD) diagnosis according to the AMPD (research aim 1)

The overall model predicting PD diagnosis according to the AMPD by time was statistically significant (Wald χ^2^ (1) = 6.4, *p* = 0.011). The probability of fulfilling PD diagnosis significantly decreased over the two years (OR = 0.54, *p* = 0.011).

### Longitudinal change in the degree of impairments in identity, self-direction, empathy, and intimacy according to the AMPD (research aim 2)

**Fig. 1:**
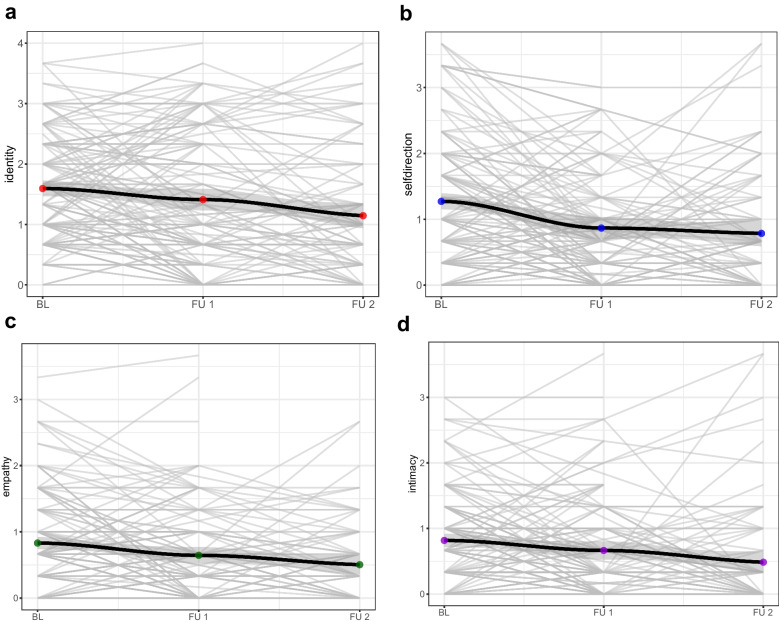
Course of Personality Functioning Elements over Two Years. The x-axes indicate the mean score and the y-axes the measurement points: BL = baseline,FU1 = Follow-up 1, FU2 = Follow-up 2. The grey area represents the confidence intervals(CI) and the points the respective means. The light grey lines show the individual courses.

The models predicting impairments in identity (Wald χ^2^ (1) = 14.46, *p* < 0.001), self-direction (Wald χ^2^ (1) = 25.83, *p* < 0.001), empathy (Wald χ^2^ (1) = 19.86, *p* < 0.001) and intimacy (Wald χ^2^ (1) = 10.15, *p* = 0.001) by time were statistically significant. As illustrated in Fig. 1, identity (β = −0.191, *p* < 0.001), self-direction (β = −0.243, *p* < 0.001), empathy (β = −0.147, *p* < 0.001) and intimacy (β = −0.136, *p* < 0.001) significantly improved over the two years. There was no evidence that the improvement rate differed between the elements (χ^2^ (3) = 4.51, *p* = 0.211; for full LGCM results, see Table S1).

### Predictors of PD remission (research aim 3a)

We have no evidence that the likelihood of PD diagnosis remission over time was moderated by age and sex (LR- χ^2^ = 3.41, *p* = 0.182), psychiatric comorbidity (LR- χ^2^ = 9.06, *p* = 0.337), psychosocial functioning (OR = 1.02, *p* = 0.447), treatment setting and dose (LR- χ^2^ = 5.65, *p* = 0.463). Corresponding null model estimates are presented in Table 2. The complete results from the alternative models for all predictors, regardless of LRT significance, are reported in Supplementary Table S2.

**Table 2:** Results of mixed regression analyses. Null models (excluding interaction terms) are reported for the variables age, sex, treatment setting, treatment dose, and psychiatric diagnoses, as the likelihood ratio test (LRT) indicated that including the interaction “predictor x time” did not significantly improve model fit compared to the null model containing only the main effects. For psychosocial functioning, the alternative model including the interaction of time and psychosocial functioning) is reported

Outcome	Model fit	Variable(s)	OR	*p*	95% CI
	Wald χ^2^(3) = 11.45, *p* = 0.01*	Time	0.55	0.014*	[−1.086, −0.122]
PD		Age	1.11	0.567	[−0.251, 0.459]
		Sex	0.10	0.013*	[−4.048, −0.474]
	Wald χ^2^ (9) = 29.23, *p* < 0.001*	Time	0.54	0.010*	[−1.093, −0.147]
		**Psychiatric diagnoses**^**1**^			
		F1	3.31	0.015*	[0.232, 2.160]
		F2	0.65	0.940	[−1.187, 1.099]
		F3	1.71	0.269	[−0.415, 1.487]
		F4	5.53	0.002*	[0.605, 2.815]
		F5	1.31	0.701	[−1.099, 1.634]
		F9	3.97	0.005*	[0.414, 2.345]
	Wald χ^2^ (7) = 24.46, *p* < 0.001*	Time	1.02	0.031*	[−1.212, −0.056]
		Psychosocial functioning^2^	0.88	< 0.001*	[−0.194, −0.073]
		(Psychosocial functioning) # (time)	1.02	0.447	[−0.027, 0.061]
	Wald χ^2^ (7) = 10.30, *p* = 0.172	Time	0.76	0.370	[−0.874, 0.326]
		**Treatment setting and dose**^**3**^			
		Inpatient dose	1.001	0.849	[−0.010, 0.012]
		Outpatient dose	1.02	0.195	[−0.008, 0.040]
			**β (SE)**		
Identity	Wald χ^2^ (3) = 34.92, *p* = < 0.001*	Time	−0.186 (0.052)	< 0.001*	[−0.288, −0.084]
		Age	0.096 (0.037)	0.010*	[0.022, 0.169]
		Sex	−0.644 (0.152)	< 0.001*	[−0.941, −0.347]
	Wald χ^2^ (9) = 138.42, *p* = < 0.001*	Time	−0.197 (0.053)	< 0.001*	[−0.300, −0.093]
		**Psychiatric diagnoses**			
		F1	0.205 (0.106)	0.053	[−0.003, 0.413]
		F2	0.099 (0.136)	0.463	[−0.166, 0.365]
		F3	0.408 (0.098)	< 0.001*	[0.217, 0.599]
		F4	0.458 (0.102)	< 0.001*	[0.257, 0.659]
		F5	0.130 (0.158)	0.410	[−0.180, 0.441]
		F9	0.364 (0.099)	< 0.001*	[0.170, 0.559]
	Wald χ^2^ (7) = 102.61, *p* = < 0.001*	Time	−0.165 (0.059)	0.005*	[−0.281, −0.050]
		Psychosocial functioning	−0.032 (0.004)	< 0.001*	[−0.340, −0.023]
		(Psychosocial functioning) # (time)	0.010 (0.004)	0.712	[−0.007, 0.010]
	Wald χ^2^ (7) = 67.35, *p* = < 0.001*	Time	−0.202 (0.070)	0.004*	[−0.339, −0.066]
		**Treatment setting and dose**			
		Inpatient dose	0.002 (0.001)	0.137	[−0.006, 0.004]
		Outpatient dose	0.005 (0.003)	0.043*	[0.0002, 0.010]
Self-direction	Wald χ^2^ (3) = 31.17, *p* = < 0.001*	Time	−0.230 (0.050)	< 0.001*	[−0.322, −0.138]
		Age	0.043 (0.037)	0.254	[−0.031, 0.116]
		Sex	−0.356 (0.150)	0.018*	[−0.649, −0.062]
	Wald χ^2^ (9) = 69.20, *p* = < 0.001*	Time	−0.239 (0.048)	< 0.001*	[−0.332, −0.145]
		**Psychiatric diagnoses**			
		F1	0.072 (0.115)	0.534	[−0.154, 0.297]
		F2	−0.113 (0.147)	0.444	[−0.401, 0.176]
		F3	0.199 (0.106)	0.061	[−0.009, 0.407]
		F4	0.343 (0.111)	0.002*	[0.125, 0.561]
		F5	−0.065 (0.171)	0.703	[−0.402, 0.271]
		F9	0.370 (0.108)	0.001*	[0.157, 0.582]
	Wald χ^2^ (7) = 80.79, *p* = < 0.001*	Time	−0.242 (0.052)	< 0.001*	[−0.344, −0.139]
		Psychosocial functioning	−0.031 (0.005)	< 0.001*	[−0.040, −0.022]
		(Psychosocial functioning) # (time)	0.008 (0.004)	0.033*	[0.001, 0.016]
	Wald χ^2^ (7) = 19.03, *p* = 0.008*	Time	−0.130 (0.064)	0.040*	[−0.255, −0.006]
		**Treatment setting and dose**			
		Inpatient dose	0.0005 (0.001)	0.712	[−0.002, 0.003]
		Outpatient dose	−0.0004 (0.003)	0.901	[−0.006, 0.005]
Empathy	Wald χ^2^ (3) = 20.69, *p* = < 0.001*	Time	−0.142 (0.033)	< 0.001*	[−0.207, −0.774]
		Age	0.028 (0.029)	0.333	[−0.029, 0.085]
		Sex	−0.120 (0.118)	0.308	[−0.350, 0.111]
	Wald χ^2^ (9) = 59.86, *p* = < 0.001*	Time	−0.147 (0.033)	< 0.001*	[−0.213, −0.082]
		**Psychiatric diagnoses**			
		F1	−0.022 (0.090)	0.806	[−0.199, 0.155]
		F2	0.044 (0.115)	0.705	[−0.183, 0.270]
		F3	−0.039 (0.083)	0.641	[−0.201, 0.124]
		F4	0.368 (0.087)	< 0.001*	[0.198, 0.539]
		F5	−0.038 (0.135)	0.776	[−0.303, 0.226)
		F9	0.282 (0.085)	0.001*	[0.116, 0.448]
	Wald χ^2^ (7) = 61.91, *p* = < 0.001*	Time	−0.113 (0.367)	0.002*	[−0.185, −0.041]
		Psychosocial functioning	−0.018 (0.004)	< 0.001*	[−0.025, −0.011]
		(Psychosocial functioning) # (time)	−0.002 (0.003)	0.558	[−0.007, 0.004]
	Wald χ^2^ (7) = 26.41, *p* = < 0.001*	Time	−0.130 (0.040)	0.001*	[−0.209, −0.052]
		**Treatment setting and dose**			
		Inpatient dose	0.001 (0.0009)	0.106	[−0.0003, 0.003]
		Outpatient dose	−0.002 (0.002)	0.247	[−0.006, 0.001]
Intimacy	Wald χ^2^ (3) = 23.37, *p* = < 0.001*	Time	−0.126 (0.042)	0.003*	[−0.209, −0.044]
		Age	0.110 (0.031)	< 0.001*	[0.050, 0.171]
		Sex	−0.194 (0.125)	0.121	[−0.438, 0.051]
	Wald χ^2^ (9) = 72.06, *p* = < 0.001*	Time	−0.140 (0.041)	0.001*	[−0.230, −0.060]
		**Psychiatric diagnoses**			
		F1	−0.042 (0.093)	0.651	[−0.226, 0.141]
		F2	0.043 (0.120)	0.721	[−0.192, 0.277]
		F3	0.109 (0.086)	0.206	[−0.060, 0.278]
		F4	0.405 (0.090)	< 0.001*	[0.228, 0.582]
		F5	0.031 (0.140)	0.823	[−0.242, 0.305]
		F9	0.255 (0.088)	0.004*	[0.083, 0.427]
	Wald χ^2^ (7) = 91.60, *p* = < 0.001*	Time	−0.130 (0.045)	0.004*	[−0.218, −0.042]
		Psychosocial functioning	−0.025 (0.004)	< 0.001*	[−0.032, −0.018]
		(Psychosocial functioning) # (time)	0.001 (0.003)	0.860	[−0.006, 0.007]
	Wald χ^2^ (7) = 34.88, *p* = < 0.001*	Time	−0.126 (0.053)	0.017*	[−0.229, −0.022]
		**Treatment setting and dose**			
		Inpatient dose	0.0004 (0.0009)	0.663	[−0.001, 0.002]
		Outpatient dose	0.001 (0.002)	0.442	[−0.002, 0.005]

OR = odds ratio, β = Coefficient, SE = Standard Error, p = p-value, 95% CI = confidence interval, PD = Personality Disorder

^1^Two patients had missing values in diagnoses, n = 225

^2^There were two missing values, n = 225

^3^Treatment dose (inpatient and outpatient) was assessed at follow-up retrospectively. Only patients that took part at each assessment (baseline, follow-up 1 and 2) were included for analysis. One patient was excluded because a work-integration-centre was wrongly interpreted as an inpatient clinic, n = 67

### Predictors of change in impairments in identity, self-direction, empathy, and intimacy (research aim 3b)

We have no evidence that age and sex moderated the degree of improvement in identity (LR- χ2 = 0.52, *p* = 0.773), self-direction (LR- χ2 = 0.22, *p* = 0.895), empathy (LR- χ2 = 3.38, *p* = 0.184) and intimacy (LR- χ2 = 2.06, *p* = 0.357) Psychiatric comorbidity did also not influence the degree of improvement in identity (LR- χ2 = 13.44, *p* = 0.098), self-direction (LR- χ2 = 11.38, *p* = 0.181), empathy (LR- χ2 = 6.94, *p* = 0.543) and intimacy (LR- χ2 = 9.96, *p* = 0.268). Further, psychosocial functioning at baseline did not affect the degree of improvement in any element except for self-direction, with adolescents with a higher level of psychosocial functioning showing less improvement over time (β = 0.008, *p* = 0.033). Finally, treatment setting and dose did not influence the degree of improvement in identity (LR- χ2 = 7.19, *p* = 0.304), self-direction (LR- χ2 = 4.00, *p* = 0.677), empathy (LR- χ2 = 9.22, *p* = 0.162) and intimacy (LR- χ2 = 9.89, *p* = 0.130). For psychosocial functioning, results from the alternative model are presented in Table 2; for all other predictors Table 2 shows the null model estimates. Full results from the alternative models for all predictors are available in Supplementary Table S2. 

## Discussion

The present study examined changes in PD diagnoses and the degree of personality dysfunction according to the AMPD—including potential differences in the rates of improvement across the elements – over two years in an adolescent patient cohort recruited from a specialized outpatient clinic for early intervention for PD. Additionally, we explored if age, sex, psychiatric comorbidity and psychosocial functioning at baseline as well as treatment setting and dose influenced these changes. The following main findings emerged: First, the probability for fulfilling a PD diagnosis according to the AMPD significantly decreased over two years, and second, there were significant improvements in identity, self-direction, empathy and intimacy, with no differences in the magnitude of improvement between the elements. These changes were of small-to-moderate size. Third, age, sex, psychiatric comorbidity and psychosocial functioning at baseline, as well as treatment setting and therapy dose did not impact the likelihood of PD diagnosis remission or the degree of improvement in personality dysfunction, except that higher levels of psychosocial functioning at baseline were associated with less improvement in self-direction.

Our findings align with research on adolescent patients with personality pathology using a traditional categorical/symptom approach according to DSM-5 section II, which reported remission of PD or a reduction in PD symptoms [31, 52, 53]. Moreover, the results are consistent with recent research on PF conducted in clinical cohorts of adults and youth [29, 30], which, in contrast to this study, relied on self-report measures to assess PF. Taken together, this study contributes to the growing evidence that PD in adolescents are not intractable and can be improved with early intervention, and extends previous research by being the first to demonstrate this using an interview-based measure of severity of personality dysfunction rather than traditional Section II PD measure. The absence of differences in the magnitude of improvement between the elements of PF may reflect their interconnected nature, where progress in one area can simultaneously enhance others, leading to similar patterns of improvement. Indeed, the DSM-5 articulates Level of Personality Functioning as a unidimensional severity criterion and factor analytic work supports this conceptualization especially when bifactor models are used to evaluate its factor structure [54, 55]. Although this study was not a randomized controlled trial (RCT), which limits our ability to attribute the improvements in PF solely to treatment, the findings are noteworthy. The majority of participants received treatment over two years and evidence suggests that, in the absence of intervention, PD symptoms tend to increase rather than decrease during adolescence [31, 56, 57].

Contrary to our hypothesis, neither of the examined variables (i.e., age, sex, psychiatric comorbidity, and psychosocial functioning at baseline, and treatment setting and dose) influenced the likelihood of PD diagnosis remission or the degree of improvement in personality dysfunction, except that participants with higher psychosocial functioning at baseline were found to show less improvement in self-direction after two years. One potential explanation for this finding is that individuals with higher psychosocial functioning may have had minimal or no impairments in PF at baseline, leaving less room for measurable improvement. Indeed, our results show that adolescents with higher psychosocial functioning exhibited significantly lower self-direction impairments at baseline (as reported in Table 2), supporting this interpretation. However, the observed effect of psychosocial functioning at baseline on improvement in self-direction needs replication, as it was considerably small.

The absence of an effect of sex on the change in PD diagnosis and the degree of personality dysfunction according to the AMPD over time could be explained by the unbalanced sex ratio in our sample, with the majority being female. In addition, the absence of an influence of age, psychiatric comorbidity, as well as treatment setting and therapy on the likelihood of PD diagnosis remission or the degree of improvement in personality dysfunction – which stands in contrast to what has been observed in traditional categorical/symptomatic PD research [12, 28] – could be attributed to the fact that PF and traditional categorical PD diagnoses measure different constructs. PF captures an overall adaptability across various contexts on a broad and continuous scale, while categorical diagnoses (according to DSM-5 section II) use specific criteria to classify individuals into distinct categories [23]. An alternative explanation for the lack of an effect of age on the change in PD diagnosis and the degree of personality dysfunction over time could be that age in our sample was not equally distributed, as most patients were at the older end of the scale and the proportion of younger patients was much smaller. Moreover, although this study was observational, the treatment received by most of the participants may have enhanced general resilience and adaptive functioning by improving interpersonal skills and emotional regulation [39], thereby reduced the impact of comorbid psychopathology on PF trajectories. Additionally, while prior studies have shown a link between treatment duration and PD diagnosis/symptom reduction [36, 37], this was not the case in our study, suggesting that longer and therefore more expensive treatments are not necessarily needed. Finally, from a statistical perspective, one could argue that a larger sample is necessary to detect moderating effects. However, all these explanations remain speculative, as no studies have yet examined the influence of age, sex, psychiatric comorbidity, psychosocial functioning, and treatment setting and dose on changes in personality dysfunction, and thus comparison with existing literature remains limited.

Overall, our study demonstrates that PF impairments according to the AMPD are sensitive to change, contributing to the growing body of evidence supporting that personality pathology is not as intractable as previously thought [12, 28]. Clinically, dimensional assessments of PD pathology provide practical advantages, as they are more sensitive to subtle improvements compared to categorical PD assessments [18, 58, 59], capture changes across multiple domains, offer richer information [60] to inform treatment planning and guide tailored interventions [27, 61], and focus on core personality impairments rather than transient acute symptoms, which may provide a more clinically meaningful picture of patient functioning [62]. In our study, the rates of improvement across the four elements were comparable, supporting the claim that PF is a unidimensional construct [63, 64]. However, empirical findings on the factor structure of measures assessing PF have been mixed [65], calling for further research on the dimensionality of the level of PF construct.[66]. Moreover, clinically, the individual element scores may still provide valuable information for treatment planning, as they allow clinicians to identify specific areas of impairment that may benefit from targeted interventions. Additionally, further research is needed to examine individual trajectories based on PF, as this would allow moving beyond group-level findings and identifying patterns of change unique to specific subgroups or individuals, enabling more personalized and targeted interventions. Future studies should also conduct RCTs to evaluate the effectiveness of existing therapies such as DBT-A or MBT on PF as primary outcome measure.

Clear strengths of the present study are the large clinical cohort recruited from a specialized clinic for adolescents with PD pathology and its longitudinal design, which allows for the examination of changes in personality dysfunction over an extended period. We also employed a structured interview to assess PF, which represents the gold standard to assess PD pathology. However, some important limitations also need to be considered. First, we did not conduct a RCT and thus, we cannot conclude that the observed improvements are solely related to the treatments received. Additionally, it needs to be taken into account that although we have a clinical cohort, a small subset of patients did not receive any treatment. Second, reasons for dropout were not systematically recorded, which limits our ability to analyse potential factors influencing retention. Third, the majority of participants were female, which is typical in clinical settings, but limits the generalisability to males and the potential to identify sex differences in PF improvements. Fourth, treatment dose was assessed retrospectively through patient self-report, which may limit the reliability of the data, and information about the type of treatment patients received was not obtained. In addition, cumulative treatment dose could only be calculated for participants who completed both follow-up assessments, which reduced the sample size and limits the generalizability of the finding. Fifth, we assessed personality dysfunction solely through a clinical interview, whereas a multi-informant approach incorporating the perspectives of clinicians, patients, and parents would be preferable. Lastly, only a fifth of the sample met full PD criteria according to the AMPD and STiP-5.1 mean scores remained below the clinical threshold of two across elements, with higher values for self- functioning than for interpersonal functioning. The relatively lower interpersonal scores may reflect the developmental stage of our sample, as identity formation is likely a key driver of PD development at this age [21, 55, 67]. However, this also limits the generalizability of our findings to adolescents with more severe personality functioning impairments, particularly in the interpersonal domain.

## Conclusion

This study demonstrates that PF impairments according to the AMPD among adolescent patients can change over time. This indicates that personality dysfunction is relatively malleable and can be improved with early intervention, independent of age, sex, and psychiatric comorbidity. However, further research is needed to understand individual trajectories, as this would provide valuable insights into the different patterns of change and enable the development of more personalized interventions tailored to the specific patterns of PF impairments of each patient.

## Supplementary Information

Below is the link to the electronic supplementary material.

Supplementary file1 (DOCX 47 KB)

## Data Availability

Data are available upon request from the corresponding author.
