# Correction: Personality functioning improvements in adolescents from an early intervention clinic for personality disorders

**DOI:** 10.1007/s00787-025-02941-0

**Published:** 2026-01-13

**Authors:** Luana Palermo, Marialuisa Cavelti, Silvano Sele, Carla Sharp, Corinna Reichl, Michael Kaess

**Affiliations:** 1 University Hospital of Child and Adolescent Psychiatry and Psychotherapy, University of Bern, Bern, Switzerland; 2 Graduate School for Health Sciences, University of Bern, Bern, Switzerland; 3 Department of Psychology, University of Houston, Houston, TX USA; 4 Department of Child and Adolescent Psychiatry, Centre for Psychosocial Medicine, University Hospital Heidelberg, Heidelberg, Germany

**Correction: European Child & Adolescent Psychiatry**

10.1007/s00787-025-02913-4

In the original version of this article, the second paragraph in the Results section under the subheading Predictors of change in impairments in identity, self-direction, empathy, and intimacy (research aim 3b) should be removed.

And the table 1 should appeared as

**Table:** 

	Baseline (*n* = 227)	Follow-up 1 (*n* = 118)	Follow-up 2 (*n* = 87)
**Family status n(%)**			
Living together	106 (46.70)		
Parents separated/divorced	115 (50.66)		
Parents separated by death	2 (0.88)		
Parents never lived together	3 (1.32)		
Unknown/other	1 (0.44)		
**School type** **n(%)**			
No school-leaving qualification	7 (3.08)		
Primary school (ISCED levels 0–1; at least 6 school years)	23 (10.13)		
Secondary school (ISCED level 2; 9–10 school years)	166 (73.13)		
High school (ISCED level 3; 12–13 school years)	28 (12.33)		
Other/ no information	3 (1.32)		
**Diagnoses**^***1***^ ***n(%)***			
F1 Mental and behavioral disorders due to psychoactive substance use	77 (33.92)		
F2 Schizophrenia, schizotypal and delusional disorders	32 (14.22)		
F3 Affective disorder	138 (61.06)		
F4 Neurotic, stress-related and somatoform disorders	141 (62.39)		
F5 Behavioural syndromes associated with physiological disturbances and physical factors	21 (9.29)		
F6 Personality and behavioural disorders^2^	40 (17.62)		
F9 Behavioural and emotional disorders with onset in childhood and adolescence	100 (44.25)		
**STiP-5.1** ***M*** **(SD)**			
Total score	1.13 (0.66)	0.90 (0.73)	0.73 (0.72)
Identity	1.60 (0.85)	1.41 (1.16)	1.15 (1.07)
Self-direction	1.27 (0.98)	0.87 (0.85)	0.79 (0.92)
Empathy	0.83 (0.72)	0.65 (0.71)	0.51 (0.61)
Intimacy	0.82 (0.76)	0.67 (0.77)	0.49 (0.81)
PD n(%)	56 (24.67)	20 (16.95)	12 (13.79)
**Treatment** ***n (%)***			
Number of participants with inpatient treatment in the past year^3^		29 (26.13)	20 (25.64)
Of those, inpatient days in the past year M(SD)		13.12 (38.93)	21.67 (52.64)
Number of participants with outpatient treatment in the past year^4^		94 (80.34)	57 (65.52)
Of those, outpatient sessions attended in the past year M(SD)^5^		19.5 (13.32)	31 (27.92)

*M* = mean, *SD* = standard deviation, *n* = sample, *ISCED* = International Standard Classification of Education STiP-5.1 = Semi-Structured Interview for Personality Functioning, PD = Personality Disorder

^1^F0 (organic mental disorders), F7 (mental retardation) and F8 (disorders of psychological development) were not assessed. Comorbidity was possible. Two patients had missing values in each F category

^2^ F6 is represented by Borderline Personality Disorder assessed with the Structured Interview for DSM-5 Borderline Personality Disorder

^3^ There were some missing values and one patient was excluded because reported inpatient clinic was not a psychiatric facility: For Follow-up 1 *n* = 111, for Follow-up 2 *n* = 78

^4^ There was one missing value in Follow-up 1, *n* = 117

^5^There was one missing value in Follow-up 2, *n* = 56

The original article has been corrected.

