# Supplementary material for: Personality functioning improvements in adolescents from an early intervention clinic for personality disorders
Source: Eur Child Adolesc Psychiatry. 2025 Nov 10;35(4):1119–31. doi: 10.1007/s00787-025-02913-4 (PMC13219104; doi:10.1007/s00787-025-02913-4)
Supplement: Supplementary file 1 — Supplementary file1 (DOCX 47 KB) [file 787_2025_2913_MOESM1_ESM.docx]

**Supplementary Material to**

*“****Personality Functioning Improvements in Adolescents from an Early Intervention Clinic for Personality Disorders”***

Luana Palermo, Marialuisa Cavelti, Silvano Sele, Carla Sharp, Corinna Reichl, Michael Kaess

Content:

S1 Table: Latent Growth Curve Model Estimates for Changes in Elements

S2 Table: Mixed-effects regression results of the Alternative Models:

Table S1 Latent Growth Curve Model Estimates for Changes in Elements

| **Outcome** | **Coefficient** | **SE** | **z** | **p** | **95% CI** | |
| --- | --- | --- | --- | --- | --- | --- |
| Identity | -0.190 | 0.045 | -4.21 | <.000 | -0.278 | -0.101 |
| Self-direction | -0.235 | 0.047 | -4.99 | <.000 | -0.328 | -0.143 |
| Empathy | -0.142 | 0.033 | -4.30 | <.000 | -0.207 | -0.077 |
| Intimacy | -0.139 | 0.038 | -3.62 | <.000 | -0.214 | -0.063 |

Notes. SE= Standard Error, z= test statistic, p= p-value, 95% CI = confidence interval

Table S2 Mixed-effects regression results of the Alternative Models

| **Outcome** | **Model fit** | **Variable(s)** | **OR** | ***p*** | **95% CI** |
| --- | --- | --- | --- | --- | --- |
| PD | Wald χ^2^(5) = 12.74, *p*= .026 | Time | 0.49 | .011 | [-1.28, -0.163] |
|  |  | Age | 1.27 | .243 | [-0.161, 0.634] |
|  |  | Sex | 0.10 | .025 | [-4.264, -0.288] |
|  |  | (age) # (time) | 0.71 | .081 | [-0.733, 0.043] |
|  |  | (sex) # (time) | 0.95 | .953 | [-1.926, 1.815] |
|  | Wald χ^2^ (17) = 25.46, *p*= .085 | Time | 0.23 | .146 | [-2.838, 0.421] |
|  |  | **Psychiatric diagnoses^1^** |  |  |  |
|  |  | F1 | 4.525 | .015 | [0.288, 2.731] |
|  |  | F2 | 0.931 | .923 | [-1.516, 1.374] |
|  |  | F3 | 2.280 | .171 | [-0.356, 2.004] |
|  |  | F4 | 4.006 | . 037 | [0.082, 2.694] |
|  |  | F5 | 2.242 | . 339 | [-0.849, 2.463] |
|  |  | F9 | 3.934 | .022 | [0.2, 2.538] |
|  |  | (F1) # (time) | 0.563 | .347 | [-1.770, 0.622] |
|  |  | (F2) # (time) | 1.228 | .761 | [-1.119, 1.530] |
|  |  | (F3) # (time) | 0.618 | .390 | [-1.577, 0.616] |
|  |  | (F4) # (time) | 2.984 | .145 | [-0.375, 2.562] |
|  |  | (F5) # (time) | 0.228 | .193 | [-3.706, 0.7481] |
|  |  | (F9) # (time) | 1.363 | .596 | [-0.835, 1.455] |
|  | Wald χ^2^ (7) = 24.46, *p*<.001 | Time | 0.53 |  |  |
|  |  | Psychosocial functioning^2^ | 0.88 | <.001 | [-0.194, -0.073] |
|  |  | (Psychosocial functioning) # (time) | 1.02 | .447 | [-0.027, 0.061] |
|  | Wald χ^2^ (13) = 11.15, *p*=.598 | Time | 0.39 | .193 | [-2.366, 0.477] |
|  |  | **Treatment setting and dose^3^** |  |  |  |
|  |  | Inpatient dose | 1.00 | .982 | [-0.141, 0.014] |
|  |  | Outpatient dose | 1.01 | .439 | [-0.019, 0.044] |
|  |  | (Inpatient dose) # (time) | 1.00 | .774 | [-0.007, 0.010] |
|  |  | (Outpatient dose) # (time) | 1.01 | .515 | [-0.013, 0.027] |
|  |  |  | **β (SE)** |  |  |
| Identity | Wald χ^2^ (5) = 35.51, *p*= <.001 | Time | -0.169 (0.058) | .004 | [-0.282, -0.055] |
|  |  | Age | 0.096 (0.039) | .015 | [0.019, 0.0172] |
|  |  | Sex | -0.605 (0.161) | <.001 | [-0.921, -0.289] |
|  |  | (Age) # (time) | 0.001 (0.039) | .989 | [-0.075, 0.076] |
|  |  | (sex) # (time) | -0.099 (0.141) | .483 | [-0.375, 0.177] |
|  | Wald χ^2^ (17) = 153.83, *p*= <.001 | Time | -0.191 (0.113) | .091 | [-0.413, 0.031] |
|  |  | **Psychiatric diagnoses** |  |  |  |
|  |  | F1 | 0.245 (0.115) | .032 | [0.021, 0.470] |
|  |  | F2 | 0.226 (0.147) | .125 | [-0.063, 0.514] |
|  |  | F3 | 0.428 (0.105) | <.001 | [0.221, 0.634] |
|  |  | F4 | 0.389 (0.111) | <.001 | [0.173, 0.606] |
|  |  | F5 | 0.244 (0.172) | .156 | [-0.093, 0.581] |
|  |  | F9 | 0.336 (0.107) | .002 | [0.126, 0.547] |
|  |  | (F1) # (time) | -0.093 (0.120) | .438 | [-0.329, 0.142] |
|  |  | (F2) # (time) | -0.355 (0.154) | .021 | [-0.657, -0.053] |
|  |  | (F3) # (time) | -0.048 (0.108) | .658 | [-0.260, 0.164] |
|  |  | (F4) # (time) | 0.186 (0.113) | .098 | [-0.035, 0.407] |
|  |  | (F5) # (time) | -0.338 (0.179) | .059 | [-0.689, 0.013] |
|  |  | (F9) # (time) | 0.081 (0.112) | .468 | [-0.139, 0.302] |
|  | Wald χ^2^ (7) = 102.61, *p*= <.001 | Time | -0.165 (0.059) | .005 | [-0.280, -0.050] |
|  |  | Psychosocial functioning | -0.032 (0.004) | <.001 | [-0.340, -0.023] |
|  |  | (Psychosocial functioning) # (time) | 0.010 (0.004) | .712 | [-0.007, 0.010] |
|  | Wald χ^2^ (13) = 75.10, *p*= <.001 | Time | -0.174 (0.140) | .216 | [-0.449, 0.101] |
|  |  | **Treatment setting and dose** |  |  |  |
|  |  | Inpatient dose | 0.0004 (0.001) | .764 | [-0.002, 0.003] |
|  |  | Outpatient dose | 0.006 (0.003) | .066 | [-0.004, 0.111] |
|  |  | (Inpatient dose) # (time) |  |  |  |
|  |  | (Outpatient dose) # (time) |  |  |  |
| Self-direction | Wald χ^2^ (5) = 31.41, *p*= <.001 | Time | -0.237 (0.053) | <.001 | [-0.340, -0.133] |
|  |  | Age | 0.051 (0.041) | .219 | [-0.321, 0.132] |
|  |  | Sex | -0.371 (0.170) | .030 | [-0.704, -0.036] |
|  |  | (Age) # (time) | -0.016 (0.034) | .643 | [-0.083, 0.051] |
|  |  | (sex) # (time) | 0.025 (0.128) | .843 | [-0.223, 0.275] |
|  | Wald χ^2^ (17) = 81.38, *p*= <.001 | Time | -0.190 (0.103) | .066 | [-0.392, 0.013] |
|  |  | **Psychiatric diagnoses** |  |  |  |
|  |  | F1 | 0.111 (0.132) | .403 | [-0.149, 0.370] |
|  |  | F2 | 0.001 (0.170) | .999 | [-0.333, 0.333] |
|  |  | F3 | 0.329 (0.121) | .007 | [0.090, 0.568] |
|  |  | F4 | 0.218 (0.128) | .088 | [-0.324, 0.469] |
|  |  | F5 | 0.056 (0.199) | .779 | [-0.334, 0.445] |
|  |  | F9 | 0.355 (0.124) | .004 | [0.112, 0.598] |
|  |  | (F1) # (time) | -0.050 (0.110) | .649 | [-0.268, 0.167] |
|  |  | (F2) # (time) | -0.189 (0.143) | .187 | [-0.471, 0.091] |
|  |  | (F3) # (time) | -0.206 (0.010) | .038 | [-0.401, -0.011] |
|  |  | (F4) # (time) | 0.1964 (0.103) | .058 | [-0.006, 0.399] |
|  |  | (F5) # (time) | -0.187 (0.166) | .260 | [-0.511, 0.138] |
|  |  | (F9) # (time) | 0.020 (0.104) | .846 | [-0.184, 0.225] |
|  | Wald χ^2^ (7) = 80.79, *p*= <.001 | Time | -0.242 (0.052) | <.001 | [-0.344, -0.139] |
|  |  | Psychosocial functioning | -0.031 (0.005) | <.001 | [-0.040, -0.022] |
|  |  | (Psychosocial functioning) # (time) | 0.008 (0.004) | .033 | [0.001, 0.016] |
|  | Wald χ^2^ (13) = 23.24, *p*= .039 | Time | -0.142 (0.131) | .279 | [-0.398, 0.115] |
|  |  | **Treatment setting and dose** |  |  |  |
|  |  | Inpatient dose | 0.0004 (0.002) | .775 | [-0.004, 0.003] |
|  |  | Outpatient dose | -0.002 (0.004) | .949 | [-0.007, 0.007] |
|  |  | (Inpatient) # (time) | 0.001 (0.001) | .303 | [-0.001, 0.003] |
|  |  | (Outpatient) # (time) | -0.0001 (0.002) | .949 | [-0.004, 0.004] |
| Empathy | Wald χ^2^ (5) = 24.31, *p*= <.001 | Time | -0.115 (0.037) | .002 | [-0.188, -0.043] |
|  |  | Age | 0.030 (0.315) | .341 | [-0.032, 0.092] |
|  |  | Sex | -0.022 (0.130) | .868 | [-0.276, 0.023] |
|  |  | (Age) # (time) | -0.003 (0.024) | .889 | [-0.050, 0.044] |
|  |  | (sex) # (time) | -0.156 (0.089) | .079 | [-0.330, 0.018] |
|  | Wald χ^2^ (17) = 66.94, *p*= <.001 | Time | -0.124 (0.072) | .087 | [-0.266, 0.018] |
|  |  | **Psychiatric diagnoses** |  |  |  |
|  |  | F1 | -0.036 (0.101) | .723 | [-0.234, 0.162] |
|  |  | F2 | 0.006 (0.130) | .964 | [-0.249, 0.260] |
|  |  | F3 | -0.087 (0.093) | .350 | [-0.269, 0.095] |
|  |  | F4 | 0.382 (0.098) | <.001 | [0.191, 0.573] |
|  |  | F5 | 0.048(0.152) | .754 | [-0.250, 0.345] |
|  |  | F9 | 0.325 (0.095) | .001 | [0.139, 0.510] |
|  |  | (F1) # (time) | 0.005 (0.778) | .951 | [-0.148, 0.157] |
|  |  | (F2) # (time) | 0.070 (0.101) | .483 | [-0.127, 0.267] |
|  |  | (F3) # (time) | 0.095 (0.067) | .175 | [-0.042, 0.231] |
|  |  | (F4) # (time) | -0.024 (0.072) | .740 | [-0.166, 0.118] |
|  |  | (F5) # (time) | -0.155 (0.116) | .181 | [-0.383, 0.072] |
|  |  | (F9) # (time) | -0.084 (0.073) | .252 | [-0.227, 0.059] |
|  | Wald χ^2^ (7) = 61.91, *p*= <.001 | Time | -0.113 (0.037) | .002 | [-0.185, -0.041] |
|  |  | Psychosocial functioning | -0.018 (0.004) | <.001 | [-0.025, -0.011] |
|  |  | (Psychosocial functioning) # (time) | -0.002 (0.003) | .558 | [-0.007, 0.004] |
|  | Wald χ^2^ (13) = 36.75 *p*= <.001 | Time | -0.1333 (0.081) | .100 | [-0.292, 0.026] |
|  |  | **Treatment setting and dose** |  |  |  |
|  |  | Inpatient dose | 0.0003 (0.001) | .753 | [-0.002, 0.002] |
|  |  | Outpatient dose | -0.003(0.002) | .138 | [-0.008, 0.001] |
|  |  | (Inpatient) # (time) | 0.001 (0.001) | .096 | [-0.0001, 0.003] |
|  |  | (Outpatient) # (time) | 0.001 (0.001) | .352 | [-0.001, 0.004] |
| Intimacy | Wald χ^2^ (5) = 25.81, *p*= <.001 | Time | -0.122 (0.046) | .008 | [-0.213, -0.031] |
|  |  | Age | 0.124 (0.033) | <.001 | [0.060, 0.188] |
|  |  | Sex | -0.166 (0.135) | .219 | [-0.431, 0.098] |
|  |  | (Age) # (time) | -0.037 (0.030) | .219 | [-0.097, 0.022] |
|  |  | (sex) # (time) | -0.054 (0.112) | .628 | [-0.275, 0.166] |
|  | Wald χ^2^ (17) = 83.91, *p*= <.001 | Time | -0.185 (0.088) | .034 | [-0.357, -0.014] |
|  |  | **Psychiatric diagnoses** |  |  |  |
|  |  | F1 | -0.084 (0.104) | .418 | [-0.289, 0.112] |
|  |  | F2 | -0.063 (0.134) | .638 | [-0.326, 0.120] |
|  |  | F3 | 0.053 (0.096) | .582 | [-0.135, 0.241] |
|  |  | F4 | 0.414 (0.101) | <.001 | [0.217, 0.612] |
|  |  | F5 | 0.161 (0.157) | .302 | [-0.145, 0.468] |
|  |  | F9 | 0.304 (0.977) | .002 | [0.112, 0.495] |
|  |  | (F1) # (time) | 0.056 (0.094) | .551 | [-0.128, 0.293] |
|  |  | (F2) # (time) | 0.120 (0.121) | .099 | [-0.0373, 0.436] |
|  |  | (F3) # (time) | 0.129 (0.084) | .127 | [-0.036, 0.293] |
|  |  | (F4) # (time) | -0.022 (0.088) | .798 | [-0.194, 0.149] |
|  |  | (F5) # (time) | -0.263 (0.140) | .060 | [-0.537, 0.012] |
|  |  | (F9) # (time) | -0.107 (0.088) | .222 | [-0.280, 0.650] |
|  | Wald χ^2^ (7) = 91.60, *p*= <.001 | Time | -0.130 (0.05) | .004 | [-0.218, -0.042] |
|  |  | Psychosocial functioning | -0.025 (0.004) | <.001 | [-0.032, -0.018] |
|  |  | (Psychosocial functioning) # (time) | 0.001 (0.003) | .860 | [-0.006, 0.007] |
|  | Wald χ^2^ (13) = 46.04, *p*= <.001 | Time | -0.177 (0.105) | .092 | [-0.384, 0.029] |
|  |  | **Treatment setting and dose** |  |  |  |
|  |  | Inpatient dose | -0.001 (0.001) | .427 | [-0.030, 0.001] |
|  |  | Outpatient dose | 0.00004 (0.002) | .983 | [-0.004, 0.004] |
|  |  | (Inpatient) # (time) | 0.002 (0.001) | .049 | [0.00001, 0.003] |
|  |  | (Outpatient) # (time) | 0.002 (0.002) | .306 | [-0.002, 0.005] |

OR= odds ratio, β=Coefficient, SE= Standard Error, p= p-value, 95% CI= confidence interval, PD= Personality Disorder^1^

Two patients had missing values in diagnoses, n= 225

^2^There were two missing values, n= 225

^3^Treatment dose (inpatient and outpatient) was assessed at follow-up retrospectively. Only patients that took part at each assessment (baseline, follow-up 1 and 2) were included for analysis. One patient was excluded because a work-integration-centre was wrongly interpreted as an inpatient clinic, n= 67
